# Network dynamics in nociceptive pathways assessed by the neuronal avalanche model

**DOI:** 10.1186/1744-8069-8-33

**Published:** 2012-04-26

**Authors:** José Jiun-Shian Wu, Hsi-Chien Shih, Chen-Tung Yen, Bai-Chuang Shyu

**Affiliations:** 1 Institute of Zoology, National Taiwan University, Taipei, Republic of China; 2 Institute of Biomedical Science, Academia Sinica, Taipei, Republic of China

**Keywords:** Pain assessment, Neuronal avalanche, Network dynamics

## Abstract

**Background:**

Traditional electroencephalography provides a critical assessment of pain responses. The perception of pain, however, may involve a series of signal transmission pathways in higher cortical function. Recent studies have shown that a mathematical method, the neuronal avalanche model, may be applied to evaluate higher-order network dynamics. The neuronal avalanche is a cascade of neuronal activity, the size distribution of which can be approximated by a power law relationship manifested by the slope of a straight line (i.e., the α value). We investigated whether the neuronal avalanche could be a useful index for nociceptive assessment.

**Findings:**

Neuronal activity was recorded with a 4 × 8 multichannel electrode array in the primary somatosensory cortex (S1) and anterior cingulate cortex (ACC). Under light anesthesia, peripheral pinch stimulation increased the slope of the α value in both the ACC and S1, whereas brush stimulation increased the α value only in the S1. The increase in α values was blocked in both regions under deep anesthesia. The increase in α values in the ACC induced by peripheral pinch stimulation was blocked by medial thalamic lesion, but the increase in α values in the S1 induced by brush and pinch stimulation was not affected.

**Conclusions:**

The neuronal avalanche model shows a critical state in the cortical network for noxious-related signal processing. The α value may provide an index of brain network activity that distinguishes the responses to somatic stimuli from the control state. These network dynamics may be valuable for the evaluation of acute nociceptive processes and may be applied to chronic pathological pain conditions.

## Findings

The critical evaluation of the pain response, especially in chronic, spontaneous pain, is valuable for clinical treatment. Traditional pain assessment measures the patient’s waveform pattern, frequency domain, and pair-wise cross-correlations of electroencephalography (EEG) and magnetoencephalography (MEG) recordings [1,2] to characterize the pain response [3,4]. The perception of pain, however, may involve a series of signal transmission pathways in higher cortical function [5,6]. Recent studies have shown that a mathematical model, the neuronal avalanche model, can estimate higher-order cortical network dynamics [7]. This model shows a cascade of neuronal activity in neuronal networks, the neuronal avalanche event size distribution of which can be approximated by a power law distribution manifested by the slope of the α value [8]. This distribution can be taken as an index of network dynamics, with an α value in the range of −1 to −2 *in vitro* and *in vivo *[8-11]. This phenomenon is robust and lasts for many hours in continuous recordings in rats [9], cats [11], and monkeys [10]. Calculating the power law exponent could offer quantitative means to evaluate the efficacy and the state of cortical networks for information transmission [7-12], which could not be provided by conventional multi-electrode recording methods. Recent studies have shown that the neuronal avalanche exists under many physiological conditions, such as wakefulness, slow-wave sleep, and rapid eye movement sleep, and may be involved in larger amounts of information processing [12]. However, direct evidence that correlates network dynamics with functional tasks associated with specific signal processing, such as nociceptive responses, is still lacking. Nociceptive information was conveyed in lateral and medial pain pathways which project to the primary somatosensory cortex (S1) and anterior cingulate cortex (ACC) respectively [13-15]. The ACC receives mainly inputs from the medial thalamus and play a different pain-related functional role from that of the S1 [16,17]. Thus the medial and lateral pain systems provide two distinct nociceptive pathways for testing the differential nociceptive responses to peripheral noxious stimuli. Thus the aim of the present study was to examine whether the neuronal avalanche in nociceptive pathways can objectively indicate network activity in brain regions that process nociceptive information.

**Figure 1:**
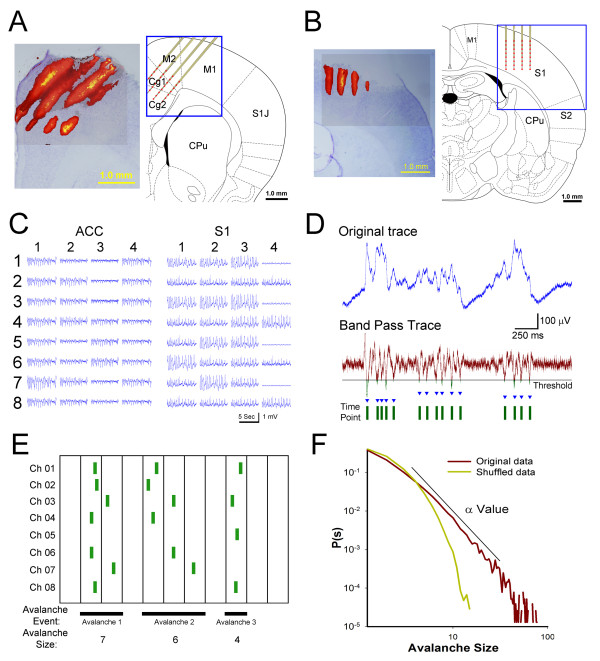
** Recording in the ACC and S1*in vivo*and calculation of the neuronal avalanche.** (A) The recording site of the Michigan probe was placed in the ACC at an angle. The electrode tracks were visible as red Dil florescence that overlaid Nissl-stained histology. (B) The recording probe in the S1 was inserted vertically. The electrode tracks were visible as red Dil florescence that overlaid Nissl-stained histology. (C) Eight recording traces in four tracks are displayed in a typical example recorded in the ACC and S1, respectively. (D) The original electrophysiological recording was filtered to preserve high-frequency unit activity. The time-point was selected as the nLFP that exceeded four-times the standard deviation of the threshold of basal activity. (E) The nLFP time-points were counted from each channel and framed in 4 ms time bins. Each avalanche event is defined as the continuous selective nLFP response until a blank frame occurred. The involved channel numbers in each avalanche event were summed as the avalanche size. (F) The distribution of the avalanche size with occurrence probability is plotted on a log-log scale. A neuronal avalanche that has a fitted straight-line slope of the α value indicates a power law relationship. Shuffled data were calculated from raw recording data.

The recording positions of 4 × 8 multichannel electrode probes in the anterior cingulate cortex (ACC; Figure 1A) and primary somatosensory cortex (S1; Figure 1B) were verified by examining the DiI that was coated on the probe and left red fluorescence on the electrode tracks in the coronal section. To maximize the recording regions and the estimation of network activities, some electrodes in the multichannel probe may cover adjacent M2 brain region which has been shown to involve in nociceptive transmission [18]. Data obtained from M2 region were also included for neuronal avalanche calculation. The 32 channel traces of spontaneous oscillations of extracellular field potentials were recorded under continuous isoflurane anesthesia (Figure 1C). These negative local field potentials (nLFP) have been described in previous studies as neuron population depolarization [8]. The time-point selected from the nLFP of each channel at which the nLFP exceeded the threshold was marked as an active digital unit (Figure 1D). These digital time-points were collected from all 32 channels for the avalanche size calculation. For example (Figure 1E), the avalanche size of the activity from eight recording electrodes was the summation of the involved electrodes with active units in continuous timeframes. The distribution of the avalanche size was plotted on a log-log scale. A straight line fit the power law distribution with the slope of the α value (Figure 1F). The same dataset was shuffled and plotted on a log-log scale for a comparison with the power law distribution data. This procedure confirmed that the original data that was collected time-sequentially was interdependent and ordered in time.

**Figure 2:**
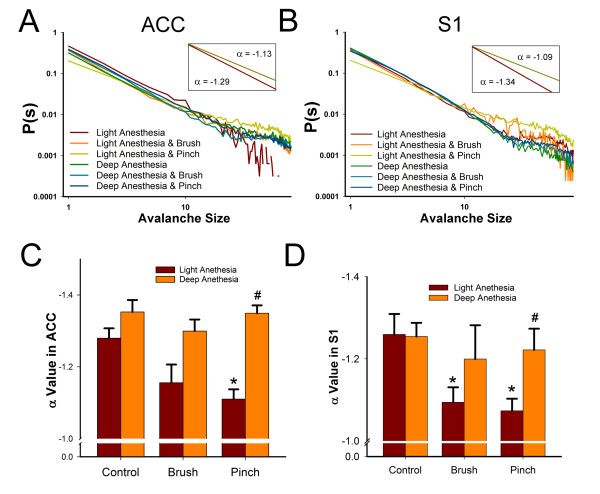
** Comparison of the neuronal avalanche of nociceptive responses in the ACC and S1 under light and deep anesthesia.** (A) Colored lines indicate the distribution of avalanche size to probability in the ACC. The X-Y axis is on a log-log scale. The α values of the control condition (red) and pinch stimulation condition (light green) under light anesthesia are shown in the inset. (B) Neuronal avalanche size distribution in the S1. The α values in the control condition (red) and pinch stimulation condition (light green) under light anesthesia are shown in the inset. (C) The bar graph shows the significant change in the α value in the ACC during pinch stimulation under light anesthesia, and this effect was reversed under deep anesthesia. *p < 0.05, pinch stimulation *vs*. control under light anesthesia; ^#^*p* < 0.05, pinch stimulation under light anesthesia *vs*. pinch stimulation under deep anesthesia. (D) The bar graph shows the significant change in the α value in the S1 during pinch stimulation under light anesthesia, and this effect was reversed under deep anesthesia. *p < 0.05, pinch or brush stimulation *vs*. control under light anesthesia; ^#^*p* < 0.05, pinch or brush stimulation under light anesthesia *vs*. pinch or brush stimulation under deep anesthesia.

The cortical network activity in the ACC and S1 showed a power law distribution. The α values were between −0.991 to −1.585 in all of the experiments. Our previous study has shown that the nociceptive responses in the ACC have higher threshold than that in the S1 [17]. Thus the differential responses to innocuous brush, and noxious pinch stimuli in these two regions were used to test the relative effects on the network activities. A typical example of the α slope under light and deep anesthesia during control (non-stimulation), brush, and pinch stimulation in the ACC and S1 is illustrated in Figure 2A and B, respectively. All data set were shuffled to test the interdependence of neuronal events. For clarity, the shuffled data were not shown. Although the spontaneous oscillations under deep anesthesia were characterized by intermittent quiescent periods, no significant difference was found in the α value between light and deep anesthesia under control conditions (ACC: -1.279 ± 0.027 under light anesthesia *vs*. -1.273 ± 0.123 under deep anesthesia, *n* = 6; S1: -1.259 ± 0.050 under light anesthesia *vs*. -1.251 ± 0.042 under deep anesthesia, *n* = 6). Under light anesthesia when compared with the control group, pinch stimulation increased the α values in the ACC and S1 (Figure 2A, B, inset), and this increase was significant (*p* < 0.05; Figure 2C, D) under light anesthesia. Brush stimulation also significantly increased the α values in the S1 (Figure 2D) but not in the ACC (Figure 2C) under light anesthesia. Under deep anesthesia, the increase in α values induced by pinch and brush stimulation was blocked in both the ACC (Figure 2C) and S1 (Figure 2D).

**Figure 3:**
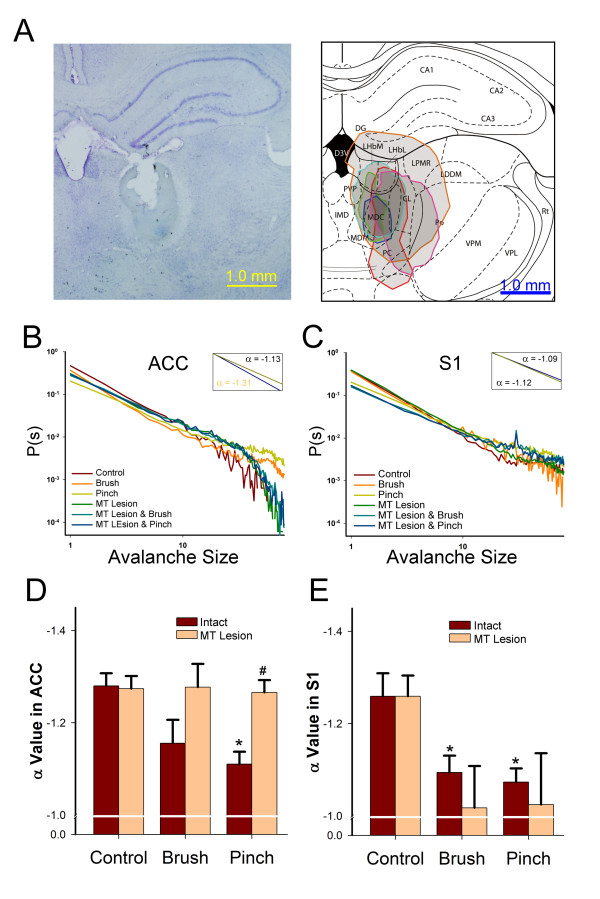
** Effect of MT lesion on neuronal avalanche in ACC and S1.** (A) A typical lesion site of the MT is shown in the left panel. The lesion sites in five different experiments were overlapped to show the overall distributions in the MT. (B) Colored lines indicate the distribution of avalanche size to probability in the ACC. The α values of pinch stimulation with an intact MT (red) and pinch stimulation with MT lesion (light green) are shown in the inset. (C) Colored lines indicate the distribution of avalanche size to probability in the S1. The α values of pinch stimulation with an intact MT (red) and pinch stimulation with MT lesion (blue) are shown in the inset. (D) The bar graphs show that pinch stimulation increased the α value compared with control in the ACC, but this effect was blocked by MT lesion. *p < 0.05, pinch stimulation *vs*. control under light anesthesia; ^#^*p* < 0.05, pinch stimulation with an intact MT *vs*. pinch stimulation with MT lesion. (E) The bar graphs show that both brush and pinch stimulation increased the α value compared with control in the S1. The MT lesion did not influence this effect. The intact groups in D and E are the same data set of light anesthesia groups in the Figure 2C and 2D respectively. *p < 0.05, pinch or brush stimulation *vs*. control under light anesthesia. No significant difference was found between pinch or brush stimulation with an intact MT *vs*. pinch or brush stimulation with MT lesion.

To examine whether the network dynamics were modulated by a specific pathway, the medial thalamus (MT) was lesioned (Figure 3A), in which the relay nociceptive inputs to the ACC were severed and the inputs to the S1 remained intact. The α value significantly increased in response to both brush and pinch stimulation in the S1 but was not affected by MT lesion (Figure 3C, E, inset). The MT lesion blocked the significant increase in α values in the ACC during pinch stimulation (Figure 3B, D, inset).

The present study showed that the neuronal avalanche could be detected and modulated in nociceptive-reactive brain regions. The power law distribution indicated that the cortical network for pain signaling was in a critical state and optimal for a large amount of signal processing [7-10,19]. The neuronal avalanche, which has been noted as a state of self-organized criticality (SOC), may be an intrinsic property of cortical networks that results from the interactions between neurons in local circuits [7]. Modeling studies have suggested that SOC networks are optimized for input processing, information storage, and transfer [20-22]. Thus, the neuronal networks in the ACC and S1 operate in a critical state and maintain a moderate level of interactions that can satisfy the competing demands of nociceptive information capacity and transmission [8,12,19]. The present study had three major findings. First, the neuronal avalanche revealed large changes in network dynamic, reflected by changing the noxious inputs. The slope of the power law distribution revealed the state of the network dynamics in which increasing α values indicated the increasing possibility of larger activity events and an expansion of the field of excitatory networks [9]. Noxious inputs may alter the excitation-to-inhibition ratio in the cortical network and thus change the network dynamics profile and modulate information processing [12]. Second, network dynamics may be modulated by the level of anesthesia. Isoflurane affects many types of neuronal transmitters in the brain, such as acetylcholine and 5-hydroxytryptamine in the prefrontal cortex and glutamate and γ-aminobutyric acid in the somatosensory cortex [23]. Thus, deep anesthesia may downregulate the excitatory inputs of cortical dynamics and causes a shift of α values. Third, the modulation of the neuronal avalanche is input-specific and pathway-dependent. The α value increased in the ACC in response to pinch stimulation and was affected by MT lesion, but not in the S1. Ascending nociceptive responses were blocked by MT lesion, and the network dynamics in the ACC reverted to control values. The noxious stimulation-induced increase in α values in the ACC was blocked by MT lesion, whereas the brush and pinch stimulation-induced increases in α values in the S1 were not affected by MT lesion. These results indicate that the network dynamics that are modulated by noxious inputs are pathway-specific.

The sensitivity of this analytical method can distinguish changes in cortical network dynamics following peripheral somatic stimulation. We recently used an animal model of central pain and found hyperexcitability and allodynia [24] that may change cortical dynamics by inducing thalamocortical dysrhythmia. The thalamic rhythm is an essential element in the generation and perception of neuropathic pain, and dysrhythmia of these activities can disturb the synchronicity of pain processing [25]. Abnormal neuronal activities could be subtracted from network dynamics by calculating the neuronal avalanche in the altered network operations. We anticipate that the neuronal avalanche model may provide an objective index of network dynamics for the evaluation the abnormal cortical rhythmicity and occurrence of spontaneous pain in chronic pathological conditions, such as neuropathic and central pain.

## Material and Methods

### Preparation of animals and electrodes

Male Sprague–Dawley rats (300–400 g) were housed in an air-conditioned room with free access to food and water. All of the experiments were performed in accordance with the guidelines of the Academia Sinica Institutional Animal Care and Utilization Committee. The rats were initially anesthetized with 4 % isoflurane (in pure O_2_) in an acrylic box. The animals were then placed in a stereotaxic apparatus and maintained under anesthesia with 2 % isoflurane during surgery. One Michigan probe with 32 contact points (150 μm-lead interval, eight leads on one shank, and four parallel shanks) was used to record the extracellular field potentials in the right ACC. Another Michigan probe was used to record extracellular field potentials in the hindpaw projection area in the right S1. DiI was dissolved in isopropanol at a saturated concentration and coated on the Michigan probe three times to ensure successful coating. The animals were subsequently maintained under anesthesia with 1.25-2.5 % isoflurane during the recording session. The depth of anesthesia was continuously monitored by an anesthesia monitor (Capnomac Ultima, General Electric Company, Fairfield, CT, USA) that monitored the minimum alveolar concentration (MAC) of isoflurane. Two depths of anesthesia, light and deep, were maintained, based on three criteria: (*i*) isoflurane MAC value for light anesthesia and 2×MAC for deep anesthesia; (*ii*) the presence (light anesthesia) or absence (deep anesthesia) of a withdrawal response to pinch stimulation of the paw; (*iii*) an EEG waveform and FFT peak value that shifted from 5 Hz (light anesthesia) to 2 Hz (deep anesthesia). To deactivate the MT, a tungsten electrode was inserted into the MT for electrolytic lesion, which was performed with a direct current of 100 μA for 100 s by a constant current pulse generator (Model 2100, A-M Systems, WA, USA).

### Data analysis

All of the raw LFP data were recorded for 22 min and filtered with a preamplifier with a 0.1 Hz to 20 KHz band pass. The sampling rate of the recorded analog signals was 40 kHz, and the data were processed using a multichannel data acquisition system (ME, Multi Channel Systems, Reutlingen, Germany) on a computer. The nLFP data were further processed by filtering at 10–200 Hz. The time-point was selected as the nLFP that exceeded four-times the standard deviation of the threshold of basal activity and was marked as the digital unit for further neuronal avalanche calculation. The filtered traces were searched for time points at which they reach the threshold. The processed data thus contained a serial time point of nLFP and could be framed by selected time-bin. The time-bin was set to 4 ms according to our previous study [26]. Each avalanche size is defined as number of digitized unit in each frame and the frame numbers of various neuronal avalanche sizes were counted for the avalanche size distribution. The avalanche size distribution was calculated using Matlab software (The MathWorks, Natick, MA, USA) and plotted using SigmaPlot software (Systat Software, Chicago, IL, USA). The slope of the power law distribution was selected from 1 to 10 in avalanche size, with a fitting index, R^2^, lager than 0.9. To verify that the power law distribution of unit activity is interdependent events, the original events were randomized to produce shuffled data. A fitting index of the shuffled data less than 0.9 indicated a change from a power law distribution to a Poisson distribution. The comparison of α values between groups was made using Student’s *t*-test and STSS software (IBM SPSS, IL, USA). Values of *p* < 0.05 were considered statistically significant.

## Competing interests

The authors declare that they have no competing interests.

## Author contributions

JJSW and HCS participated in the design of the study, conducted the experiments, analyzed the data, and drafted the manuscript. CTY participated in the discussion of the experimental results and made experimental suggestions. BCS conceived of the study, participated in its design and coordination, and participated in the writing of the manuscript. All of the authors read and approved the final manuscript.
